# Associations between hair cortisol concentration, income, income dynamics and status incongruity in healthy middle-aged women

**DOI:** 10.1016/j.psyneuen.2016.02.008

**Published:** 2016-05

**Authors:** Bianca Serwinski, Gyöngyvér Salavecz, Clemens Kirschbaum, Andrew Steptoe

**Affiliations:** a Psychobiology Group, Department of Epidemiology and Public Health, University College London, 1-19 Torrington Place, London WC1E 6BT, UK; b Institute of Behavioral Sciences, Semmelweis University, Nagyvárad tér 4, H-1089 Budapest, Hungary; c Department of Psychology, Technische Universität Dresden, Germany

**Keywords:** Hair cortisol, Stress biomarkers, Income, Education, Status incongruity

## Abstract

A body of research demonstrates that financial disadvantage is associated with general health inequalities and higher mortality rates. Most studies make use of cross-sectional analyses, although income can also be viewed as a dynamic concept. The use of endocrine-markers as proxies for health can provide information about the pathways involved in these associations. Hair cortisol analysis has been developed as a method for assessing sustained cortisol output as it provides an estimate of cumulative cortisol secretion over a prolonged time. The present study assessed income and income trajectory over a 4-year period in 164 working women (aged 26–65) in relation to hair cortisol in a longitudinal design. A negative association between hair cortisol and concurrent income was found (*p* *=* 0.025) and hair cortisol and changes in income over 4 years (*p* < 0.001), after adjustment for age, BMI, smoking status, hair treatment and country. Status incongruity, a mismatch between educational status and income group, was related to higher cortisol levels compared with status congruity (*p* = 0.009). These findings suggest that psychoneuroendocrinological pathways might partially explain the relationship between lower socio-economic status and adverse health outcomes. Future longitudinal research using hair cortisol analysis is warranted to clarify the time course of social mobility in relation to long-term cortisol, to investigate other underlying psychosocial factors implicated in these associations, and to determine the exact health implications of the neuroendocrine perturbations in individuals with limited economic resources.

## Highlights

• We examined associations of income and income dynamics with hair cortisol.
• We found a gradient negative relationship between hair cortisol and income.
• A negative change in income was related to higher hair cortisol.
• Social incongruity (a mismatch in education and income) affected hair cortisol.
• Our finding supports a relative income effect, but not an absolute income effect.

## 1 Introduction

During the past three decades, widening income inequality in many developed countries has become a major problem. Highlighting the importance of socio-economic factors in relation to health, research in various European countries demonstrates that financial disadvantage leads to many types of health inequality, including higher mortality and morbidity rates (Marmot, 2002). Financial stability is a critically important life domain as many essential daily activities and opportunities for education, realisations and achievements are dependent on existing financial resources. Self-report measures of health outcomes and status have often been used to evaluate the link between income and health, but they might underestimate the true negative socioeconomic inequalities of health (Baker et al., 2004). Research has shown that there can be striking discrepancies between subjectively reported health outcomes and objectively measured biological markers or health conditions, and that education and income may contribute to erroneous reporting of health outcomes (Johnston et al., 2009, Mackenbach et al., 1996). The use of more objective measures, such as endocrine, metabolic or immune markers as proxies for health may improve the reliability of such findings and provide information about the pathways mediating these associations.

Most studies have examined income as a static phenomenon, making use of cross-sectional data (Gunasekara et al., 2011). However, income status can also be regarded as a dynamic entity, and the effect of changes over time might further inform the relationship between accumulated economic adversity, exposure to stressors and health. Cumulative socioeconomic disadvantage and social downward mobility over the life course have been shown to be related to cardiovascular disease mortality, but the underlying pathways between these factors are poorly understood (Johnson-Lawrence et al., 2015). Further, changes in the labour market, including job insecurity and downward social mobility, have created the phenomenon of status incongruity. People whose occupational position is lower than might be expected from their educational attainment and those whose status is greater than might be expected from their education can be regarded as experiencing status incongruity. Status incongruity has long been established to be linked to health outcomes (Braig et al., 2011). Longitudinal studies making use of objective biomarkers offer the possibility of investigating the effects of cumulative socioeconomic disadvantage, income trajectories and status incongruity on health.

One mechanism potentially linking socioeconomic status (SES) and health is the psychoneuroendocrine pathway and the involvement of stress-related cortisol responses (Lupien et al., 2001). A body of research has suggested a link between lower income and higher salivary and urinary cortisol levels (Dowd et al., 2009, Jimenez et al., 2015). Lower income and education have also been associated with elevated diurnal cortisol values and with flatter diurnal rhythms in a graded fashion (Cohen et al., 2006). But although low income has been found to be predominantly associated with elevated cortisol, there are contradictory findings, partly due to variations in methodology and cortisol assessments, i.e. cortisol reactivity levels, diurnal levels or morning values (Dowd et al., 2009).

Psychoneuroendocrinological research has predominantly used saliva, blood and urine for cortisol assessment. Assays of cortisol from these sources reflect momentary cortisol concentrations at the time of sampling rather than sustained levels and are influenced by timing, the collection method and handling plus a variety of situational factors such as diet, sleep patterns, environmental stressors, acute psychological states and participant adherence (Hansen et al., 2008, Kudielka et al., 2003). In the last decade, there has been a steep rise in studies using hair as a source for cortisol analyses, with many results mirroring findings from the saliva cortisol literature and thus demonstrating its utility as a measure of HPA axis function (Raul et al., 2004, Stalder and Kirschbaum, 2012, Staufenbiel et al., 2013). Hair cortisol has been associated with numerous adversities or stress-related conditions, such as pregnancy (D'Anna-Hernandez et al., 2011), chronic pain (Van Uum et al., 2008), dementia caregiving (Stalder et al., 2014) or unemployment (Dettenborn et al., 2010), with chronic illnesses, such as diabetes (Feller et al., 2014), asthma (Kamps et al., 2014) and also obesity (Veldhorst et al., 2014). Studies relating hair cortisol with psychiatric illnesses and perceived stress have produced somewhat inconsistent results (Herane Vives et al., 2015, Sharpley et al., 2012, Staufenbiel et al., 2013).

The relationship between financial status (income) and hair cortisol is not well studied. The only study to date reported elevated hair cortisol in participants earning less than the minimum wage in different communities in sub-Saharan Africa (Henley et al., 2014). Some mixed evidence also exists for the effect of parental income on children’s hair cortisol levels (Bosma et al., 2015). While one study found support for a negative association between parental income and pre-schooler’s hair cortisol levels, another study found lower maternal and paternal education but not income per se to be linked with elevated hair cortisol levels, supporting the notion that different measures of SES might associate differently and that methodological variations lead to heterogeneous findings (Henley and Koren, 2014, Vaghri et al., 2013). Trajectories over time in income have not been studied at all, although our group previously showed changes in salivary cortisol in relation to changes in financial strain over 3 years (Steptoe et al., 2005). The protective effect of upward social mobility on different health outcomes, including cardiovascular disease mortality risk, is well documented, but no study has evaluated this phenomenon in relation to hair cortisol. There is therefore a sound rationale for exploring socioeconomic factors in relation to this long-term cortisol marker. The aims of the study were therefore to investigate hair cortisol concentration in relation to concurrent income, income change over a 4 year period, education and status incongruity in a healthy sample of women. We hypothesised that lower income and a negative shift in income over four years would predict higher hair cortisol levels, and that lower education and status incongruity would also show an association with elevated hair cortisol.

## 2 Method

### 2.1 Participants, procedure and study design

The Daytracker Study involved healthy working women aged 26–65 years employed at the University College London (UCL) and neighbouring institutions in London and at Semmelweis University in Budapest. The study involved assessment of a range of psychosocial factors, emotional experience and biomarkers in two contrasting cultures, and data were collected in 2007–2008. Participants were re-contacted via email, telephone, LinkedIn and Facebook four years later in 2012 for the present follow-up study by the study researchers at both sites. Exclusion criteria were pregnancy, chronic or acute medical conditions, e.g. cardiovascular disease or cancer, or regular intake of steroid medication, as these factors are known to affect cortisol secretion. Of the 199 London sample, 68 women took part in the follow-up assessment (33 were excluded due to missing baseline information or contact details and 14 because of ineligibility upon contact, 4 refused, 10 could not be scheduled and 70 were otherwise not contactable due to invalid e-mail addresses, non-delivery of e-mails or non-responsiveness). Of the 202 Budapest sample, there were 97 women who were followed-up (20 were excluded because of ineligibility upon contact, 7 refused, 11 could not be scheduled, 1 person died and 66 were not contactable). Participants did not differ from those who did not take part on any of the socio-demographic (education, income), health-related and anthropometric factors (smoking, BMI) except in age, since follow-up participants were slightly older than those who did not take part. Participants received an honorarium for their involvement, and the study was approved by relevant Research Ethics Committees in London and Budapest.

Both at baseline and at follow-up, participants reported income in 8 categories, educational attainment and demographic characteristics (age and smoking status) and anthropometric measures were recorded. At follow-up, hair samples were taken for the assessment of cortisol.

### 2.2 Hair sample collection and analyses

A scalp hair strand of 3 cm was collected from the posterior vertex position by cutting the hair as close to the scalp as possible with fine medical scissors. These were placed onto aluminium foil, stored in a dry, dark place, until shipped to the Technical University of Dresden, Germany. The wash procedure and steroid extraction were undertaken using high performance liquid chromatography– mass spectrometry (LC/MS), as described by Kirschbaum et al. (2009), with a minimum of 10 mg ± 0.5 mg of hair, cut from each 3 cm hair segment. Based on an average monthly hair growth of approximately 1 cm, the scalp-nearest hair segment of 3 cm represents averaged cortisol accumulated over an approximate timespan of three months prior to sampling. Hair-specific factors that could affect hair cortisol concentration (washing frequency, hair colour, product use and hair treatment/dyeing) were assessed by self-report.

### 2.3 Measures and statistical analyses

Data from the two study sites were combined to improve statistical power for the analyses. Income data were obtained in multiple categories in each country, but because of absolute differences in personal annual income ranges between the UK and Hungary, these variables were harmonised into low, medium and high income categories. The thresholds for the three groups in London were <£25,000, £25,000–£34,999, and >£35,000, while the corresponding cut-offs in Budapest were <1,080,000 HUF, 1,080,000–1,559,999 HUF, and >1,560,000 HUF. Educational systems also differ markedly, so level of education was classified according to whether or not the individual had a University degree. The three income categories were used in the analyses of concurrent income. Changes in income over time were defined as deterioration, no change or an improvement in income group. For this, baseline income group values (i.e. lower = 1/medium = 2/higher = 3) were subtracted from follow-up income group values (lower = 1/medium = 2/higher = 3), which resulted in five potential scores (−2/−1/0/1/2). To increase cell sizes and thus statistical power, positive values were aggregated into one positive score and negative values into one negative score, hence positive values indicate an improvement and negative values a deterioration in income; whilst a value of 0 indicates no change in income group over the 4 year period. Status incongruity was analysed with an interaction term between income (computed as a binary variable; with lower income group being <£35,000/<1,560,000 HUF and higher income group being >£35,000/>1,560,000 HUF, retrospectively, based on sample distribution) and education. Four income group/education categories emerged: low-status congruent group (lower income group; lower education group/no degree), negative incongruent group (income status < education), positive incongruent group (income status > education) and high-status congruent group (higher income group; higher education group/degree).

Hair cortisol values were positively skewed so were log transformed before analysis. One hair cortisol outlier (defined as three standard deviations above the mean) was identified and removed from statistical analyses. Socio-demographic differences and hair-related characteristics were analysed with *t*-tests and Chi^2^ tests for continuous and categorical variables retrospectively.

With regards to ethnicity, there were only 12 women in the London sample, and none in Budapest, that could be categorised as non-white. There was no significant effect on hair cortisol levels nor on income or education, and when controlling for ethnicity in the models, the results were unchanged. Including also marital status and having children did not change any associations.

Analysis of covariance (ANCOVA), including polynomial contrast analysis for linear and quadratic terms, were conducted with hair cortisol concentration as the outcome, and concurrent income group, change in income group or status incongruity in separate models. Analyses were adjusted for age, BMI, smoking and hair treatment, as these factors have been identified in the literature as covariates for hair cortisol. An additional covariate was country to control for differences among the two samples.

## 3 Results

### 3.1 Descriptive information about the study sample

The characteristics of the study sample are summarized in Table 1. Respondents were aged 43.6 on average, typically had BMIs in the normal range, and were mostly non-smokers. Forty-five percent had children and 57% were married or in a marriage-like relationship. Around 72.2% had a university degree, and half of respondents were in the higher income category. Participants in Budapest were on average 3.5 years older than those in London (*p* = 0.022), but there were no other country differences in any of the socio-demographic characteristics (BMI, income, education and smoking status) or in hair cortisol levels (all *p*’s > 0.14). There were no associations between hair cortisol and age, BMI, smoking status, marital status, having children, weekly hair washes, hair colour or use of hair products (all *p*’s > 0.08). However, hair treatment, defined as having had hair dyed in the last 3 month prior to sampling, was associated with hair cortisol; individuals with treated hair had lower cortisol levels than those with untreated hair (1.84 vs. 2.07 ln(pg/mg), *p* = 0.026).

### 3.2 Income, income change, and cortisol

**Fig. 1:**
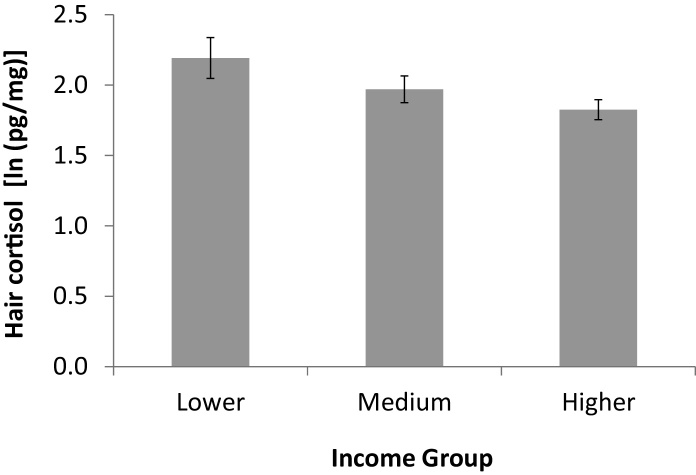
Gradient association between hair cortisol (mean log values) and concurrent income group, adjusted for age, BMI, smoking status, hair treatment and country.

**Fig. 2:**
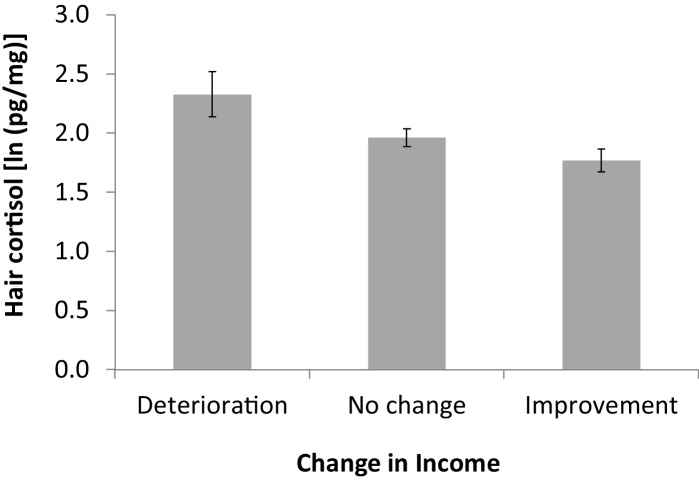
Associations between change in income group over 4 years and hair cortisol (mean log values), adjusted for age, BMI, smoking status, hair treatment and country.

There was a linear association between concurrent income group and hair cortisol levels (*p* = 0.025). Fig. 1 depicts the negative gradient between income group and hair cortisol levels, where it can be seen that cortisol concentrations were 20% higher in the lower compared with the higher income category, adjusted for age, BMI, smoking status, hair treatment and country. The analyses of income change indicated that 39.5% showed an increase in income, 53.1% reported no change, and 7.4% experienced a decrease in income over the 4 year follow-up period. There was no country by time interaction in income (*p* = 0.84). Change in income group was linearly associated with hair cortisol, with a deterioration in income group being related to higher hair cortisol levels and an improvement in income group to lower hair cortisol (*p* = 0.012), as shown in Fig. 2 (adjusted for covariates).

### 3.3 Education, status incongruity, and hair cortisol

**Fig. 3:**
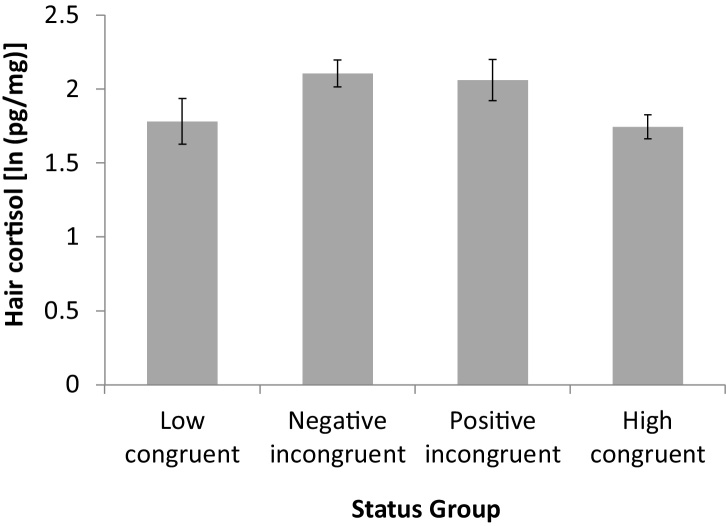
Status incongruity is associated with higher cortisol levels (mean log values) than status congruity, adjusted for age, BMI, smoking status, hair treatment and country.

Educational attainment was unrelated to hair cortisol levels (*p* = 0.82), but there was a significant interaction between income and education (*p* = 0.009). 32.6% of respondents had a lower income and greater education (negative incongruity), 14.5% had higher income despite less education (positive incongruity) and 11.6% and 41.3% reported being status congruent (low-status and high-status group, retrospectively). Fig. 3 shows how the positive and the negative incongruent groups have higher hair cortisol levels (2.07 and 1.99 ln(pg/mg), retrospectively) than the two congruent status groups (1.81 and 1.80 ln(pg/mg) for the low and high congruent group, retrospectively), adjusted for age, BMI, smoking status, hair treatment and country.

## 4 Discussion

In the present study, we found a relationship between income and income change over the last four years and hair cortisol concentrations. The pattern indicated that lower income was associated in a graded fashion with higher hair cortisol. Similarly, hair cortisol levels in individuals experiencing an unfavourable change in income over the last four years were higher than in those with no or a favourable change in income. Further, an incongruent status (a mismatch in education and income), for both over- and undereducated individuals in relation to their income group, was related to higher cortisol levels compared with a congruent status (low education/low income and high education/high income).

The results are in line with previous studies focusing on traditional cortisol specimens from saliva and blood that have reported lower income to be associated with disturbed cortisol regulation (Cohen et al., 2006, Dowd et al., 2009). It is also consistent with a relatively recent study showing that lower income as a measure of deprivation was related to higher hair cortisol levels in women from sub-Saharan African communities (Henley et al., 2014). The present study adds to these findings by showing a clear dose-response relationship between income and hair cortisol. The income measure in the present study was derived from personal salary levels. Interestingly, there are several features of low income and material deprivation that have been linked with detrimental health outcomes, namely absolute income, relative income (comparisons with relevant others at an individual level) and income inequality (comparisons with relevant others at a community level) (Miething, 2013). In the present study, the two samples did not differ in their average hair cortisol levels despite their remarkable differences in overall income; the Hungarian currency cut off points correspond to UK’s absolute income values of <£2,400, £2,400–3,600 and £3,600 + per annum. However, we found a gradient relationship *within* the different levels of income and hair cortisol after controlling for country. The associations point therefore to a strong relative income effect, but not an absolute income effect. In fact, relative income seems to have a substantial additive effect on absolute income in relation to mortality (Miething, 2013). And although income levels seem to be useful objective representations of concrete circumstances, perception of financial resources, interpretations and importance might be a significant if not an even bigger contributing factor towards ill-health. Making use of subjective assessments about income satisfaction and income perceptions in relation to context-dependent relative income might be of higher comprehensive value as they fully capture experiences and evaluations (Veenhoven, 2002).

An unfavourable change in income over the last four years was related to higher hair cortisol levels. Dynamic aspects of socio-economic and psychosocial circumstances seem to be fundamental in studying life course and disease development. In fact, one study found that income trajectory predicted cardiovascular disease mortality more than income per se, thus implicating that the dynamics, especially downward trends, have a larger impact on health than chronic financially disadvantaged ranks (Johnson-Lawrence et al., 2015).

Other than neuroendocrine pathways, studies have identified further underlying biological correlates of adverse socioeconomic circumstances, such as SNS–PNS (Sympathetic Nervous System–Parasympathetic Nervous System) dysregulation, systemic inflammation and weakened immune functions, adverse metabolic function components, along with poor health behaviours as potential mechanisms (Hickson et al., 2012, Steptoe et al., 2003). Evidence also exists linking income with various immunomarkers and cardiovascular disease risk factors (including cardiovascular disease incidence), such as leukocyte telomere length, C-reactive protein, triglyceride or fibrinogen levels; all mechanisms that also accelerate biological aging (Albert et al., 2006, Carroll et al., 2013).

The causal mechanisms of income and income dynamics and these health-related outcomes is not well understood yet, but increased stress exposure and underlying stress reactions, such as frustration, a deterioration of life quality due to limited access to resources, a disjuncture between actual and expected status and resulting feelings of deprivation are thought to mediate this relationship (Lynch et al., 2004). The present study only focused on a relatively short time period to assess income change, but provides some preliminary findings on the relationship between social mobility and neuroendocrine functioning. A further distinction between income mobility and occupational status mobility could be a fruitful area of research, as they have been shown to co-occur in a diverging or converging manner over the life-course (Breen et al., 2016). The effect of intergenerational mobility (between parent and child) on hair cortisol, considering the pivotal mediating role education might play in the ability of social mobility, also warrants further study (Goldthorpe, 2014). Hair cortisol is a biomarker that is very suitable for future longitudinal studies assessing sustained effects of socio-economic variations and adjustments.

Hair cortisol concentration was recently found to be negatively associated with education in a sample of young male adults, with education being divided into three subgroups: junior high school/high school/above high school (Boesch et al., 2015). We did not find a relationship between education and hair cortisol in the present study. However, we studied a group of relatively well educated women, employed at higher-education institutions in varying positions, in which a certain level of education is a prerequisite. This sample is not representative of the complete population. It is possible that comparing people across the whole spectrum of education would have shown more direct associations with hair cortisol. Although we did not find an effect of education on hair cortisol levels per se, when assessing education in relation to income group, an effect of status incongruity on hair cortisol emerged. In support of this, it has been suggested that various socioeconomic indicators (income, education, occupational position, social class or household area) should be assessed simultaneously as they present specific interdependent interactions and stratification of the different indices of SES has shown to have a specific additional effect on health (Torssander and Erikson, 2010).

A discrepancy between educational attainment and level of income indicates that the individual's status characteristics are not congruent, whether in an inferior (negative incongruent) or superior (positive incongruent) direction. Status incongruity, or also termed status inconsistency and status crystallization, has been studied in various contexts, ranging from sociology, economic and political environments to health implications (Braig et al., 2011, Lenski, 1954). For example, data from population cohort studies indicate that overqualified individuals have a higher cardiovascular disease risk than congruent qualified individuals, although this effect has not been observed in underqualified individuals (Braig et al., 2011, Honjo et al., 2014). In line with these findings, a recent 19-year longitudinal study found a higher mortality risk only in individuals who were over-educated in relation to their occupational status (negative incongruent) individuals but not in under-educated individuals (Garcy, 2015). The present study did not show this protective effect (i.e. lower hair cortisol levels) in the positive incongruent group, but rather hair cortisol levels that were comparable with those of the negative incongruent group.

Psychological strain and role conflict might be generated as a result of the economic compensation not corresponding to the individual’s intellectual skills and competencies. The underlying aspects for such conflict and dissatisfaction have been suggested to stem from expectancy discrepancy and cognitive dissonance (Sampson, 1963). For instance, shaming experiences but also depressive symptoms have been suggested as negative emotional consequences of status incongruity (Bracke et al., 2013, Lundberg et al., 2009). Epidemiological and especially psychobiological research would benefit from detailed distinctions between objective and subjective accounts of status incongruity in order to identify the pathways under which status incongruity affects psychoneuroendocrinological functioning.

Given the exploratory nature of the study, a number of limitations need to be acknowledged. A key limitation is that no hair samples were collected at baseline. This is because the sampling of cortisol in hair was not widely known in 2007 when this study was designed. This means that it is not known whether the differences in hair cortisol in relation to income seen at follow-up were present at the baseline as well. The sample was limited to middle-aged women in the two countries; therefore the results cannot be generalized to the wider population. The measure of income reflects personal income and not household income. This was due to a great part of the sample not being married and reporting personal household only. When marital status and also having children was included in the models, results remained unchanged. A proportion of the sample might live in households in which their personal income only makes a partial contribution, hence personal income may not be a good indication of economic resources. It would therefore be interesting to corroborate findings with household income, a more diverse sample and also to investigate potential sex differences. We studied women in this investigation, but men might be expected to be more sensitive to status incongruity since income is a strong influence on prestige and social status among men. A more pronounced association between status incongruity and hair cortisol is therefore possible. With regards to income change, a simple change score of income category was computed which made no distinction between magnitude of income change, meaning that small and large increases in income were treated the same. A bigger sample size allowing for more detailed change estimates would be more informative regarding the gradient relationship.

Further, only a subset of individuals could be re-contacted for the follow-up assessment mostly due to missing, invalid or non-updated contact information. Follow-up and non-follow-up participants did not differ in income and in any other baseline characteristics apart from age. For many younger individuals, London is a transient place and participants that could be reached are more settled inhabitants, a factor which might or might not be related to income dynamics. If the younger individuals that were lost at follow-up were to be included, a different pattern of income dynamics might have emerged which might have impacted the results.

Finally, hair cortisol differences among the different independent variables were significant but relatively small. This is not surprising, since income and status are two of many factors that might affect cortisol output. But if the differences reflect sustained variations in cortisol output over extended periods of months and years, they might contribute to differences in health risk.

To conclude, we found a dose-response association between income, income dynamics and hair cortisol and also an effect of status incongruity on hair cortisol. Quantifying the degree of these socio-economic elements appears to be relevant to health and underlying biological mechanisms. Clearly, there are multiple pathways by which socio-economic factors determines biological functioning and ultimately health; therefore future studies should focus on more comprehensive designs including macro- and microeconomic contexts, social, psychological, behavioural and biological factors.

## Contributors

All authors contributed significantly to the conception, design, analyses or interpretation of data and were involved in revising it critically for intellectual context. The submission of this paper was approved by all researchers.

## Funding

This work was supported by the ESRC (original study; number RES-177-25-005) and MRC (follow-up study; number 507601). The funding sources for this study did not influence the analyses or results presented in this manuscript or the conclusions drawn from this research. Andrew Steptoe is supported by the British Heart Foundation.

## Conflicts of interest

None of the authors have any conflicts of interest to declare related to the findings of this study.

## Figures and Tables

**Table 1:** Socio-demographic characteristics and hair cortisol of study participants.

Characteristics	Mean (SD)/frequency (%)			Group differences *p*-value
	Combined sample (*N* = 164)	London (*N* = 67)	Budapest (*N* = 97)	
Age	43.6 (9.8)	41.5 (9.3)	45.0 (10.0)	0.022
Body mass index (kg/m^2^)	24.1 (4.4)	24.1 (3.7)	24.1 (4.8)	0.95
Current smoker				0.21
Yes	21 (14.0)	4 (8.3)	17 (17.5)	
No	124 (85.5)	44 (91.7)	80 (82.5)	
Education (degree)				0.23
Yes	117 (72.2)	51 (77.3)	66 (68.8)	
No	45 (27.8)	15 (22.7)	30 (31.3)	
Personal income^a^				0.10
Low	26 (15.9)	15 (22.4)	11 (11.3)	
Medium	50 (30.5)	19 (28.4)	31 (32.0)	
High	88 (53.7)	33 (49.3)	55 (56.7)	
Status incongruity group^b^				0.26
Low	22 (13.6)	10 (15.2)	12 (12.5)	
Negative	52 (32.1)	23 (34.8)	29 (30.2)	
Positive	23 (14.2)	5 (7.6)	18 (18.8)	
High	65 (40.1)	28 (42.4)	37 (38.5)	
Hair cortisol				
(pg/mg)	8.39 (6.3)	8.52 (7.3)	8.30 (5.7)	0.83
ln(pg/mg)	1.92 (0.6)	1.90 (0.7)	1.93 (0.6)	0.75

a Income groups are equivalent to <£25,000/ £25,000–35,000/>£35,000 and <1,080,000 HUF/1,080,000–1,559,999 HUF/>1,560,000 HUF for London and Budapest, retrospectively.

b Status incongruity: Low (lower income status; lower education group/no degree), negative (income status < education), positive (income status > education), high (higher income status; higher education group/degree).
